# Finite-Size Corrections from the Subleading Magnetic Scaling Field for the Ising and Potts Models in Two Dimensions

**DOI:** 10.3390/e27040418

**Published:** 2025-04-11

**Authors:** Yihao Xu, Jesús Salas, Youjin Deng

**Affiliations:** 1 Department of Modern Physics, University of Science and Technology of China, Hefei 230026, China; yhxu@mail.ustc.edu.cn; 2 Departamento de Matemáticas, Universidad Carlos III de Madrid, Avenida de la Universidad 30, Edificio Sabatini, Leganés, 28911 Madrid, Spain; 3 Grupo de Teorías de Campos y Física Estadística, Instituto Gregorio Millán, Universidad Carlos III de Madrid, Unidad Asociada al Instituto de Estructura de la Materia, CSIC, Serrano 123, 28006 Madrid, Spain; 4 Hefei National Research Center for Physical Sciences at the Microscale, University of Science and Technology of China, Hefei 230026, China; 5 Hefei National Laboratory, University of Science and Technology of China, Hefei 230088, China

**Keywords:** Potts model, *O*(*n*) loop model, finite-size-scaling theory, subleading magnetic scaling field, Monte Carlo simulation, cluster algorithm

## Abstract

In finite-size scaling analyses of critical phenomena, proper consideration of correction terms, which can come from different sources, plays an important role. For the Fortuin–Kasteleyn representation of the *Q*-state Potts model in two dimensions, although the subleading magnetic scaling field, with exactly known exponent, is theoretically expected to give rise to finite-size-scaling analyses, numerical observation remains elusive, probably due to the mixing of various corrections. We simulate the O(*n*) loop model on the hexagonal lattice, which is in the same universality class as the $Q=n^{2}$ Potts model but has suppressed corrections from other sources and provides strong numerical evidence for the attribution of the subleading magnetic field in finite-size corrections. Interestingly, it is also observed that the corrections in small- and large-cluster-size regions have opposite magnitudes, and, for the special n=2 case, they compensate with each other in observables like the second moment of the cluster-size distribution. Our finding reveals that the effect of the subleading magnetic field should be taken into account in finite-size-scaling analyses, which was unfortunately ignored in many previous studies.

## 1. Introduction

Monte Carlo (MC) methods [1,2] constitute a very important research tool to investigate the dynamic and static properties of many systems in science and, in particular, in statistical mechanics. In the latter field, the typical setup is a *finite* system of dimension *d* and linear size *L* that undergoes a phase transition at a certain critical temperature. In most cases, MC simulation can only handle finite systems, even though phase transitions only occur in the thermodynamic limit $N=L^{d}\to \infty$. So, in addition to the statistical error inherent to any MC simulation, finite systems are frequently a source of systematic errors (an exception are MC simulations of self-avoiding random walks or other polymer models; see, e.g., [3]).

Therefore, in every MC study, one has to extrapolate the results obtained in finite lattices to the infinite-volume limit L→∞. Finite-size-scaling (FSS) theory [4,5,6] explains how thermodynamic quantities (like the magnetic susceptibility) behave close to a phase transition when we take the thermodynamic limit L→∞.

Let us consider, for simplicity, a finite physical system of dimension *d*, linear size *L*, and periodic boundary conditions. This system undergoes a continuous phase transition that is characterized (for simplicity) by a single relevant thermal field *t* (that measures the “distance” to the critical point), and an irrelevant field *u*, in the framework of renormalization group (RG) theory. The singular part of the free energy density scales like [7,8,9]

(1) $$f_{s}\left(g_{t},g_{u},L\right)\;=\;b^{-d}\;f_{s}\left(g_{t}\;b^{y_{t}},g_{u}\;b^{y_{u}},L^{-1}\;b\right)\;.$$

Here, *b* is any positive number, $g_{t}$ and $g_{u}$ are the nonlinear scaling fields associated to *t* and *u*, respectively, and $y_{t}=1/\nu >0$ and $y_{u}<0$ are the corresponding RG eigenvalues, where ν is referred to as the correlation-length exponent. Note that the inverse linear size $L^{-1}$ behaves like a relevant operator with exponent $y_{L}=1$. The nonlinear scaling fields $g_{t}$ and $g_{u}$ are analytic functions of *t*, *u*, and $L^{-1}$. In particular, $g_{t}=a_{1}\left(u,L^{-1}\right)\;t+a_{2}\left(u,L^{-1}\right)\;t^{2}+\cdots$, and $g_{u}=b_{0}\left(u,L^{-1}\right)+b_{1}\left(u,L^{-1}\right)\;t+b_{2}\left(u,L^{-1}\right)\;t^{2}+\cdots$. Guo and Jasnow [10] have argued, using the field-theoretic RG, that $g_{t}$ and $g_{u}$ do not depend on $L^{-1}$. If this is the case, then the functions $a_{i}$ and $b_{i}$ would depend solely on *u*. The full free energy density is equal to

(2) $$f\left(t,u,L\right)\;=\;f_{s}\left(g_{t},g_{u},L\right)+f_{r}\left(t,u\right)\;,$$

where the regular part $f_{r}$ is an analytic function of *t* and *u*, even at the critical point t=0 [11] (p. 101).

If we take the infinite-volume limit $L^{-1}=0$, and we choose *b* such that $b^{y_{t}}\;\left|g_{t}\right|=1$, we obtain

(3) $$f_{s}\left(g_{t},g_{u}\right)\;=\;{\left|g_{t}\right|}^{d\nu }\;f_{s}\left(\mathrm{sign}\left(g_{t}\right),g_{u}\;{\left|g_{t}\right|}^{-y_{u}\nu },0\right)\;.$$

Notice that the behavior at $t=0^{\pm }$ might be different.

We now require $b\;L^{-1}=1$, so that (1) becomes

(4) $$f_{s}\left(g_{t},g_{u},L\right)\;=\;L^{-d}\;f_{s}\left(g_{t}L^{1/\nu },g_{u}L^{y_{u}},1\right)\;.$$

At criticality (t=0), Equations (2) and (4) reduce to

(5) $$f\left(0,u,L\right)\;=\;f_{r}\left(0,u\right)+L^{-d}\;f_{s}\left(0,g_{u}L^{y_{u}},1\right)\;.$$

The leading behavior of $f_{s}$ is $L^{-d}$, and we obtain FSS corrections of order $L^{y_{u}},L^{2y_{u}},\ldots$ when we expand $f_{s}\left(0,g_{u}L^{y_{u}},1\right)$ as a power series in *u*.

We can obtain similar expressions for the internal energy and the specific heat if we differentiate Equations (2) and (4) with respect to *t* once or twice, and then take the t→0 limit. In these cases, we also have FSS corrections of order $L^{-1},L^{-2},\ldots$ due to the functions $a_{i}\left(u,L^{-1}\right)$.

The same arguments can be followed to derive the equation for the correlation length (actually, for a definition of the correlation length that makes sense in a finite system, e.g., the second-moment correlation length)

(6) $$\xi \left(t,u,L\right)\;=\;L\;F_{\xi }\left(g_{t}L^{y_{t}},g_{u}L^{y_{u}}\right)\;.$$

Given a thermodynamic quantity A(t) that diverges at the critical point (in the infinite-volume limit) like

(7) $$A\left(t\right)\;\propto {\;|t|}^{-\rho }\;,$$

We can generalize Equation (6) as follows

(8) $$A\left(t,u,L\right)\;=\;L^{\rho /\nu }\;F_{A}\left(g_{t}L^{y_{t}},g_{u}L^{y_{u}}\right)+A_{r}\left(t,u\right)\;,$$

where we have added a regular background term $A_{r}$ playing a similar role as the term $f_{r}$ in the free energy density [cf. Equation (5)].

Barring the effect of the irrelevant field $g_{u}$ in Equations (6) and (8), we see that the divergence of the bulk correlation length at criticality is smoothed out; i.e., ξ(0,L)∼O(L), and the behavior of A(t,L) becomes analytic in the scaled variable $g_{t}L^{1/\nu }$, even at the critical point t=0. FSS effects can be observed in a scaling window of width $O(L^{-1/\nu })$. Outside this window, $\left|t\right|\gg L^{-1/\nu }$, A(t,L) behaves basically like A(t). In this context, Li et al. [12] studied the crossover FSS theory, which shows that, as the critical point is approached at a slower rate with $\left|t\right|∼L^{-\lambda }$ and λ<1/ν, the FSS becomes dependent on the parameter λ.

This description can be generalized to include more relevant and irrelevant fields. In some models, we can also find marginal operators characterized by zero eigenvalues $y_{u}=0$. These fields give rise to multiplicative [13,14] and additive [15] logarithmic corrections not taken into account in the previous equations. Logarithmic corrections can also appear when there is a “resonance” between the RG eigenvalues [16], like, e.g., the two-dimensional (2D) Ising model. In this paper, we will not consider logarithmic corrections of any type.

FSS techniques allow us to compute reliable estimates of physical quantities that describe the infinite-volume limit (like the location of the critical point, and its critical exponents and universal amplitudes) solely from finite-size data. In order to achieve this goal, one needs a good ansatz based on Equation (8). At criticality, we have that [cf. (7)]

(9) $$\begin{array}{ccc} A(0,u,L) & = & L^{\rho /\nu }\;F_{A}\left(0,g_{u}L^{y_{u}}\right)+A_{r}\left(0\right)\\ & \propto & L^{\rho /\nu }\;\left(1+aL^{y_{u}}+bL^{-\rho /\nu }+\cdots \right)\;.\; \end{array}$$

It is very important to have a fairly good knowledge of the RG exponents (i.e., $y_{t}$, $y_{u}$, etc.) for the model at hand. This is provided in many 2D models by conformal field theory (CFT) [17]. It is also worth noticing that FSS corrections do depend on the observable *A*: distinct observables may have different FSS corrections because some amplitudes may vanish due to symmetries. A well-known example is the 2D Ising model. The FSS for the free energy, internal energy, and specific heat have integer exponents [18,19,20], but there is no trace of the exponent $y_{u}=-4/3$ predicted by Nienhuis [21]. In addition, some observables are expected to have a background term $A_{r}$ (e.g., the specific heat), while others (e.g., the correlation length) are not expected to have it. All these facts make the FSS analysis rather involved.

The *Q*-state Potts model [22,23,24,25] is an important model in statistical mechanics due to its simplicity, very rich phase diagram and connections with CFT [17], Coulomb-gas (CG) theory [26], and combinatorics [27], to mention only a few. In particular, when the 2D Potts model displays a continuous transition (i.e., when Q∈[0,4]), the leading and subleading RG exponents are known exactly in the thermal [cf. (22)] and magnetic [cf. (23)] sectors. This latter one appears when we add the dependence $g_{h}b^{y_{h}}$ in the free energy and correlation length [see Equations (1) and (6)].

The role of the leading ($y_{t1}$ and $y_{h1}=y_{h}$) and the subleading ($y_{t2}$) RG exponents have been considered in detail by the previous literature. However, the effect of the subleading magnetic exponent $y_{h2}$ (23) has been usually neglected. The aim of this paper is to show the effect of the subleading magnetic exponent $y_{h2}$ in the 2D Potts model.

In order to achieve this goal, we have considered, instead of the standard spin representation of the Potts model, the so-called Fortuin–Kasteleyn (FK) [28,29] [cf. (19)]. In this representation one can define new (geometric) observables, like the size of the largest FK cluster, the size of the clusters, the radius of gyration of the clusters, etc. Therefore, it is useful to introduce elements taken from percolation theory [30,31,32]. In particular, we consider the quantity n(s;p)—i.e., the number (per site) of clusters of size *s* at probability *p*—in the thermodynamic limit (L→∞)

(10) $$n\left(s;p\right)\;=\;s^{-\tau }\;F\left(\left(p-p_{c}\right)\;s^{\sigma }\right)\;,$$

where σ and τ are critical exponents and τ is usually refereed to be the Fisher exponent. We assume that *p* is close to the critical probability $p_{c}$, and *s* is large enough. At criticality, we obtain that $n\left(s;p_{c}\right)=s^{-\tau }F\left(0\right)$, and, taking into account corrections to scaling, the behavior at criticality of n(s;p) should be [33,34]

(11) $$n\left(s;p_{c}\right)\;=\;s^{-\tau }\;\left(a+b\;s^{-\Omega }+\cdots \right)\;,$$

where Ω is a correction-to-scaling exponent whose exact value is [34]

(12) $$\Omega \;=\;\frac{1}{d_{f}\;g}\;,$$

where

(13) $$d_{f}\;=\;y_{h1}\;=\;\frac{\left(2g+1)(2g+3\right)}{8g}\;,$$

is the fractal dimension of the FK clusters, and *g* is the CG coupling that parametrizes the critical (or tricritical) Potts model (see below and Section 2).

In a previous paper [34], we studied the correction-to-scaling exponent Ω for the critical and tricritical Potts models with Q=1,2,3,4. Actually, instead of directly simulating these models, we considered the related O(n) loop model on the hexagonal lattice *G* [35,36,37,38], whose partition function is given by

(14) $$Z_{\mathrm{loop}}\left(G;x,n\right)\;=\;\sum_{\left\{\ell \right\}}x^{E(\ell )}\;n^{N(\ell )}\;,$$

where the sum is over all possible nonintersecting loop configurations *ℓ* on *G*, N(ℓ) is the number of loops in the configuration, and E(ℓ) is the total length of all the loops. The parameter *n* represents the weight associated with each loop, and *x* is a fugacity that controls the weight of the loop’s length. When n,x are real positive parameters, (14) has a probabilistic interpretation.

The free energy of the O(n) loop model has been solved along two distinct curves [35,39,40]

(15) $$x_{\pm }\;=\;\frac{1}{\sqrt{2\pm \sqrt{2-n}}}\;.$$

The curve $x_{+}\left(n\right)$ is the critical curve of the O(n) loop model and separates the diluted phase from the dense phase. The other curve $x_{-}\left(n\right)$ lies within the (critical) dense phase. This loop model has a CG representation [26,41] with coupling *g* related to n∈(−2,2] as

(16) $$n\;=\;-2\;\cos\left(\pi \;g\right)\;,\;g\;\in \;\left(0,2\right]\;.$$

It is known that the RG trajectories in this model keep the parameter *n* fixed. Moreover, the points lying on $x_{+}\left(n\right)$ belong to the same universality class as the tricritical Potts model with $Q=n^{2}$ states (and g∈[1,2]). In the same fashion, the points in the dense phase [i.e., $x\in (x_{+}\left(n\right),\infty )$] all belong to the universality class of the critical Potts model with $Q=n^{2}$ states (and g∈(0,1]). This happens, in particular, to the points lying on $x_{-}\left(n\right)$. Notice that negative values of *n* can occur with the parametrization (16), but this is not a big issue as *n* is just the statistical weight of the loops in (14). In fact, the O(n) loop model with n∈(−2,0) can be studied with CFT techniques.

Simulating the O(n) loop model has several technical advantages over direct simulations of the critical and tricritical Potts models with $Q=n^{2}$: (1) for the diluted Potts model, the *exact* position of the tricritical point is not known; (2) the leading thermal and magnetic exponents for the Potts model ($y_{t1}$ and $y_{h1}$, respectively) do not appear in the loop model [26]; (3) critical slowing down (CSD) for cluster algorithms [42] is absent on $x_{-}$, and it is moderate on $x_{+}$, and (4) along the dense curve $x_{-}\left(n\right)$, the amplitudes corresponding to corrections from the subleading thermal scaling field vanish [42]. In particular, there is no trace of logarithmic corrections for n=2, corresponding to the critical 4-state Potts model [34,42].

**Figure 1:**
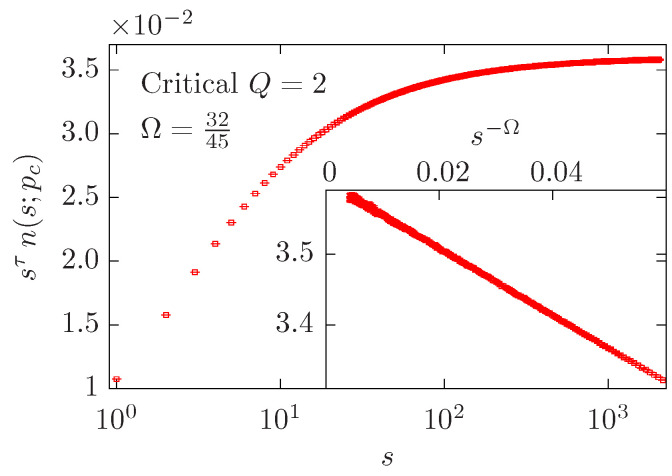
Correction-to-scaling behavior of the O(n) loop model on the dense curve $x_{-}$ (15) for $n=\sqrt{2}$; this model belongs to the universality class of the critical Ising model (Q=2). The numerical estimates were computed on a torus of linear size L=1024. The main panel depicts the quantity $s^{\tau }\;n\left(s;p_{c}\right)$ vs. *s* in the range 1≤s≲L. The inset shows the same quantity vs. $s^{-\Omega }$ (with Ω=32/45) in the range 50≲s≲L. A linear behavior is observed in agreement with Equation (11).

For each value of *n* considered in [34], we obtained a result similar to that displayed in Figure 1 for $n=\sqrt{2}$. As *s* increases, we see that the quantity $s^{\tau }\;n\left(s;p_{c}\right)$ grows and tends to a constant value ≳3.5. The transient that occurs for small and moderate values of *s* can be described by a correction-to-scaling term with an exponent Ω given by the predicted value.

Notice that, if we express the correction term of (11) in terms of a length scale $s∼R^{d_{f}}$, we obtain that this correction term becomes $a+b^{\prime }\;R^{-1/g}+\cdots$. So we expect that there is an FSS correction with an exponent $d_{f}\Omega =1/g=y_{h1}-y_{h2}$. This argument agrees with the results of Refs. [43,44]. They considered corrections to scaling of several quantities (e.g., the total mass of a FK cluster, the mass of the hull of a cluster, etc.) as a function of the radius of gyration. They found three sources of corrections: (1) the subleading thermal exponent $y_{t2}$; (2) corrections (inspired by CG arguments) with exponents $\theta^{\prime }=1/g$ (for the total mass) or $\theta^{\prime \prime }=1/\left(2g\right)$ (for the other quantities); and (3) corrections with integer exponents due to “analytic” terms.

Figure 1 clearly illustrates the effect of the subleading magnetic scaling field in the finite-cluster-size corrections for a system in the universality class of the 2D Ising model. The subleading magnetic exponent $y_{h2}$ has been computed directly by means of MCRG methods for the 2D critical Potts model [45,46,47,48] but, to the best of our knowledge, direct evidence for the existence of the corresponding FSS effects is still lacking. Moreover, for the 3D Ising model, it has been argued that the subleading magnetic field is a redundant operator [48,49,50]. For the 2D Ising model, this issue is not clear. On one side, it has also been argued that this subleading magnetic field is redundant due to the $Z_{2}$ symmetry of the model [42]. On the other side, two redundant operators have been found in the odd sector in actual MCRG simulations with 2×2 blocks and majority rule with random tie-breakers [48] and references therein. However, the corresponding eigenvalues are not compatible with $2^{y_{h2}}=2^{13/24}\approx 1.455653$ (see Table 1). Therefore, additional investigations will be needed to decide whether the subleading magnetic field is redundant or not in the 2D model.

In the present work, we have again simulated the O(n) loop model on the hexagonal lattice using the same cluster MC algorithm as in Ref. [34]. Our goal is to explore the contribution of the subleading magnetic exponent $y_{h2}$ to the finite-lattice-size corrections of that model, which we expect to follow a behavior of $L^{-1/g}$, where $1/g=y_{h1}-y_{h2}$. We have considered two geometric quantities that belong to the “magnetic” sector: the mean size of the largest cluster $C_{1}$ and the mean second moment $S_{2}$ of the critical cluster-size density $n(s,p_{c})$ (11). In fact, we have found that both quantities display an FSS term with exponent 1/g, as predicted above. In particular, for $S_{2}$ we argue that the 1/g contribution has two distinct origins. One of them corresponds to the critical cluster-size density $n(s,p_{c})$ (11), as illustrated in Figure 1 for $n=\sqrt{2}$. This part accounts for the effect of small clusters. The second origin can be attributed to the largest cluster $C_{1}$, as shown in Section 3. Based on the numerical data, these two contributions share the same correction exponent 1/g but with opposite amplitudes. This leads to certain challenges in fitting the data for $S_{2}$. Furthermore, it can be observed that for n=2 or Q=4, although both $C_{1}$ and $n(s;p_{c})$ exhibit such 1/g corrections, the 1/g correction in $S_{2}$ is absent, and the leading correction exponent is instead 2.

In our data analysis, we find that, in many cases, we have two FSS corrections with similar exponents and opposite amplitudes. This is a rather unpleasant situation for data analysis: both terms together mimic a single correction term with a rather distinct exponent. In order to disentangle these two contributions, high-precision data is needed.

The remainder of this paper is organized as follows: In Section 2, we briefly review the 2D Potts models, and in Section 3, we discuss the FSS corrections in these models. Section 4 is devoted to the description of the performed MC simulations, and in Section 5, we show our numerical findings. Finally, in Section 6, we present our conclusions.

## 2. The Potts Model

In this section, we briefly describe the “pure” and diluted Potts models in 2D. Let us start with the standard *Q*-state Potts model [22,23,24,25]. It can be defined on any finite (undirected) graph G=(V,E) of vertex set *V* and edge set *E*. In statistical mechanics, this graph is usually chosen to be a finite subset of a 2D lattice with some boundary conditions (e.g., toroidal in MC simulations). On each vertex x∈V, we place a spin $\sigma_{x}$ that can take Q∈N distinct values; that is, $\sigma_{x}\in \left\{1,2,\ldots ,Q\right\}$. Spins interact via a nearest-neighbor coupling J∈R. The Hamiltonian of this model is given by

(17) $$-\beta \;H_{\mathrm{Potts}}\;=\;J\;\sum_{\{xy\}\in E}\delta_{\sigma_{x},\sigma_{y}}\;,$$

where $\delta_{a,b}$ is the Kronecker delta function, and β is the inverse temperature. The partition function of this model is given by

(18) $$Z_{\mathrm{Potts}}\left(G;Q,J\right)\;=\;\sum_{\left\{\sigma \right\}}e^{-\beta \;H_{\mathrm{Potts}}}\;,$$

where the sum is over all possible spin configurations {σ}. We will focus on the ferromagnetic regime of this model: i.e., J>0.

At this stage, the parameters Q,J belong to N and R, respectively. We can define the *Q*-state Potts model beyond the latter ranges by using the FK [28,29] representation

(19) $$Z_{\mathrm{Potts}}\left(G;Q,v\right)\;=\;\sum_{F\subseteq E}v^{\left|F\right|}\;Q^{k(F)}\;,$$

where the sum is over all spanning subgraphs (V,F) of the graph *G*, and the temperature-like variable $v=e^{J}-1$ belongs to the physical interval v∈[0,∞) in the ferromagnetic regime. The above expression is clearly a polynomial jointly in the variables Q,v. Therefore, we can analytically promote these variables from their original physical ranges to arbitrary real, or even complex, variables. In fact, the model (19) has a probabilistic interpretation when both variables are real and satisfy Q,v>0.

The *Q*-state Potts model on any regular lattice displays a phase-transition curve $v_{c}\left(Q\right)$ in the ferromagnetic regime. This phase transition is second-order if Q∈[0,4], and first-order if Q>4 [51]. Indeed, the form of the curve $v_{c}\left(Q\right)$ depends on the lattice structure, but the critical behavior is universal. In particular, the critical *Q*-state Potts model can be represented as a CG with parameter *g* [26,41]. The relation between *Q* and *g* is given by

(20) $$\sqrt{Q}\;=\;-2\;\cos\left(\pi \;g\right)\;,\;g\in \left(0,1\right]\;.$$

Note that we find $\sqrt{Q}<0$ for g∈(0,1/2). Although this may look unphysical, it has no practical importance as the partition function (19) depends on $Q=4\cos^{2}\left(\pi g\right)\geq 0$.

A natural generalization of the above model is the diluted *Q*-state Potts model. The basic idea is to allow for vacancies in the graph *G*, which can be represented by integer variables $\tau_{x}\in \left\{0,1\right\}$ placed on the vertices of the lattice. In particular, $\tau_{x}=0$ ($\tau_{x}=1$) means that the corresponding vertex x∈V is empty (occupied). The Potts Hamiltonian (17) can be generalized in several ways. One simple form is [23,52,53]

(21) $$-\beta \;H_{\mathrm{dP}}\;=\;\sum_{\{x,y\}\in E}\tau_{x}\tau_{y}\left(K+J\delta_{\sigma_{x},\sigma_{y}}\right)-\Delta \sum_{x\in V}\tau_{x}\;.$$

In this equation, Δ plays the role of the chemical potential governing the concentration of vacancies. Other Hamiltonians have been proposed in the literature: see, e.g., [21,54,55,56,57]. In some cases, it is possible to find an FK-type representation.

The diluted Potts model appears naturally when we perform a RG transformation on the pure Potts model (17) [52,53]. In particular, the variable *Q* remains constant under RG. On the critical surface for Q∈[0,4], there is a line of attractive critical fixed points (in the same universality class as the pure Potts model), and there is another line of (repulsive) tricritical fixed points (belonging to a new universality class). Both lines meet at Q=4. For Q>4, the system renormalizes to a discontinuity fixed point (at zero temperature), as expected [58,59,60].

There is also a CG representation of the tricritical Potts model for Q∈[0,4]. In particular, the relation between *Q* and *g* is also given by (20), but the range for *g* is now g∈[1,2]. In this sense, the tricritical Potts model is the analytic extension of the critical one, or *vice versa*.

The leading and subleading thermal exponents $y_{t1}$ and $y_{t2}$ relate to *g* as [26]

(22a) $$y_{t1}=\frac{3(2g-1)}{2g},$$

(22b) $$y_{t2}=\frac{4(g-1)}{g},$$

and the corresponding magnetic exponents $y_{h1}$ and $y_{h2}$ are given by

(23) $$y_{h1}\;=\;\frac{\left(2g+1)\;(2g+3\right)}{8\;g}\;,\;\;y_{h2}\;=\;\frac{\left(2g-1)\;(2g+5\right)}{8\;g}\;.$$

The exponents (22) and (23) are displayed in Table 1 for future use. The subleading thermal exponent (22b) corresponds to the dilution operator. This one is relevant for the tricritical Potts model and irrelevant for the critical Potts model. In the critical 4-state Potts model, corresponding to g=1, that operator is marginal with $y_{t2}=0$. More precisely, the dilution operator is marginally irrelevant at Q=4. This is the origin of multiplicative [13,14] and additive [15] logarithmic corrections.

From the leading eigenvalues (22a) and (23), one can obtain the standard critical exponents: e.g.,

(24a) $$\nu =\frac{1}{y_{t1}}=\frac{2g}{3(2g-1)},$$

(24b) $$\gamma =\frac{2y_{h1}-d}{y_{h1}}\overset{d=2}{=}\frac{3+4g^{2}}{6(2g-1)}$$

and the rest can be derived using the hyperscaling relations.

## 3. Finite-Size Scaling

In percolation theory, the fraction of the largest cluster over the system volume $C_{1}/L^{d}$ acts as an order parameter, and the second moment $S_{2}$ of the cluster size distribution $n(s;p_{c})$ corresponds to the magnetic susceptibility. According to the standard FSS theory, the behavior of $C_{1}$ [cf. (33)] at criticality can be obtained by differentiating the free energy (4) with respect to the magnetic scaling field, leading to

(25) $$\begin{array}{ccc} C_{1} & = & c_{0}+a_{1}L^{y_{h1}}+a_{2}L^{y_{h2}}+\cdots \\ & \propto & L^{y_{h1}}\;\left(1+a^{\prime }L^{y_{h2}-y_{h1}}+c_{0}^{\prime }L^{-y_{h1}}\cdots \right)\;,\; \end{array}$$

where $C_{1}$ is a shorthand for $C_{1}\left(p_{c};L\right)$, and the background term $c_{0}$ comes from the analytical part of the free energy. Similarly, the FSS behavior of $S_{2}$ [cf. (34)/(35)] at criticality can be obtained by differentiating twice the free energy with respect to the magnetic scaling field, which gives

(26) $$\begin{array}{ccc} S_{2} & = & s_{0}+b_{1}L^{2y_{h1}-d}+b_{2}L^{y_{h1}+y_{h2}-d}+\cdots \\ & \propto & L^{\gamma /\nu }\;\left(1+b^{\prime }L^{-1/g}+s_{0}^{\prime }L^{-\gamma /\nu }+\cdots \right)\;, \end{array}$$

where $S_{2}$ is a shorthand for $S_{2}\left(p_{c};L\right)$, and $\gamma /\nu =2y_{h1}-d$. Hence, the first correction term should be of order $y_{h2}-y_{h1}=-1/g$ in agreement with Refs. [43,44].

The basic FSS behavior of $S_{2}$ (26) can be deduced alternatively by using Equations (11), (12) and (25)

(27) $$\begin{array}{ccc} S_{2} & = & \int_{1}^{C_{1}}s^{2}\;n\left(s;p_{c}\right)\;ds\\ & \propto & L^{\gamma /\nu }\;\left(1+b^{\prime }L^{-1/g}+c^{\prime }L^{-\gamma /\nu }+\cdots \right)\;, \end{array}$$

where we have made use of the relations $\tau =1+d/d_{f}$ and $\gamma /\nu =2y_{h1}-d$. In other words, the *leading* FSS behavior of $S_{2}$ can be obtained by differentiating the free energy or integrating the cluster-number density. Both methods yield, as expected, the same form of the leading FSS corrections $∼L^{-1/g}$. Furthermore, by using the second procedure, it is clear that the correction amplitude $b^{\prime }$ in $S_{2}$ (27) has contributions from both the largest cluster $C_{1}$ and the smaller ones [via $n(s;p_{c})$].

Let us now discuss the FSS corrections that may arise from other sources: (1) The background terms in Equations (25) and (26) ($c_{0}$ and $s_{0}$, respectively) are expected to exist, and they act effectively as correction terms with exponents $-y_{h1}$ for $C_{1}$ and $d-2y_{h1}=-\gamma /\nu$ for $S_{2}$. (2) One should have a term $\propto L^{2y_{h2}-d}$ for $S_{2}$, not explicitly shown in Equation (26), which would give a correction term with exponent −2/g. (3) For the critical Potts model, a typical source of FSS corrections is the subleading thermal scaling field, which is irrelevant and has exponent an $y_{t2}<0$ (see Table 1). Fortunately, exactly along the dense branch $x_{-}\left(n\right)$ of the O(*n*) loop model, the amplitude of the subleading thermal scaling field is exactly zero, and thus the correction term with exponent $y_{t2}$ is expected to be absent, as numerically confirmed in previous studies [42].

**Figure 2:**
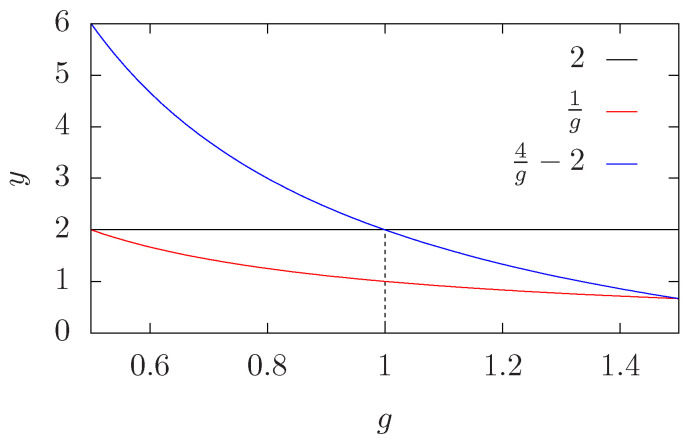
Correction-to-scaling exponents in Equation (28). We show the behavior of the different exponents appearing in Equation (28) as a function of g∈[1/2,3/2] (i.e., for Q∈[0,4]). The exponent 1/g (red curve) is the most relevant in the interval g∈[1/2,3/2]. The second most relevant exponent is 2 (black horizontal line) in the interval g∈[1/2,1], and 4/g−2 (blue curve), in the interval g∈[1,3/2].

Additional insights can be obtained by following the argument shown in Section III of Ref. [34]. Consider an annulus of size $R_{1}\times R$ with periodic (free) boundary conditions on the second (first) coordinate. When the inner radius $R_{1}$ is kept finite while the outer radius becomes asymptotically large (R≫1), the crossing probability Π(R) that a cluster connects the two boundaries is given by

(28) $$\Pi \left(R\right)\;=\;R^{d_{f}-2}\;\left(A_{0}+A_{1}\;R^{-1/g}+A_{2}\;x^{-2}+A_{3}\;R^{-(4/g-2)}+A_{4}\;R^{-(1/g+2)}+\ldots \right)\;,$$

where the dots stand for higher-order corrections and the leading correction exponent −1/g agrees with the previous analyses. Figure 2 shows the exponents in Equation (28) as a function of g∈[1/2,3/2], which corresponds to Q∈[0,4].

As shown in Ref. [34], the quantity Π(R) (28) can be related to the probability at criticality $P_{\geq R}$ that an occupied vertex is connected to an FK cluster of size greater than or equal to $s∼R^{d_{f}}$. In fact, $P_{\geq s}$ is given by the first moment of the cluster density at criticality $n(s;p_{c})$, so we expect that the FSS terms that appear in Equation (28) will also appear in the FSS behavior of both $C_{1}$ and $S_{2}$.

To summarize, let us write the FSS behavior of both $C_{1}$ and $S_{2}$ as

(29) $$A\;=\;L^{y_{A}}\;\left(a+bL^{-y_{1}}+cL^{-y_{2}}+\cdots \right)$$

where $y_{C_{1}}=y_{h1}=d_{f}$, $y_{S_{2}}=2y_{h1}-d$, and $0<y_{1}<y_{2}$ are the first two dominant exponents. Based on the above discussion, for both quantities, the leading FSS correction exponent is

(30) $$y_{1}\;=\;\frac{1}{g}\;.$$

The exact values of this exponent for the critical and tricritical Potts models are listed in Table 1. For the subdominant correction, the exponent is given by

(31) $$y_{2}=\min\left(2,\frac{4}{g}-2,y_{h1}\right)\;$$

for $C_{1}$, and

(32) $$y_{2}=\min\left(2,\frac{4}{g}-2,\frac{2}{g},2-2y_{h1}\right)\;$$

for $S_{2}$ (where d=2 has been used).

## 4. Monte Carlo Simulations

In this section, we describe the MC simulations we have performed in this work. We have used a cluster MC algorithm to simulate the O(n) loop model on both branches $x_{\pm }$ (15). Instead of simulating this model, we have used its representation as a generalized Ising model on the dual triangular lattice. We refrain from giving details of the algorithm, as it is well explained in Refs. [34,42].

As explained in the previous section, we have focused on two main physical quantities. In each iteration of the MC simulation, we recorded the sizes of the different FK clusters in the system. Let us denote $C_{k}$ as the size of the *k*th largest cluster.

The first quantity of interest is the mean size of the largest cluster $C_{1}$

(33) $$C_{1}\;=\;\langle C_{1}\rangle \;.$$

The second quantity measured is the mean second moment $S_{2}$ of the critical cluster size $n(s;p_{c})$. We first define the observable $S_{2}$

(34) $$S_{2}\;=\;\frac{1}{L^{d}}\sum_{i}C_{i}^{2}\;,$$

where the sum is over all clusters in the system. Then, $S_{2}$ is given by

(35) $$S_{2}\;=\;\langle S_{2}\rangle \;.$$

We have simulated the O(n) loop model at $n=1,\sqrt{2},\sqrt{3},2$ on the dense branch $x_{-}$. These models have the same universality class as the corresponding critical Potts model with $Q=n^{2}$ states. We have also simulated the O(n) loop model at $n=1,\sqrt{2},\sqrt{3}$ on the dilute branch $x_{+}$. In this case, these models have the same universality class as the corresponding tricritical Potts model with $Q=n^{2}$ states. For each model, the systems had linear sizes equal to L=4, 5, 6, 7, 8, 9, 10, 11, 12, 14, 16, 18, 20, 24, 28, 32, 36, 40, 48, 56, 64, 80, 96, 112, 128, and 256. For the O(n) loop model at $n=\sqrt{2},\sqrt{3}$ on the dense branch $x_{-}$, we have also considered systems of linear size L=1024. We have used periodic boundary conditions in all our simulations. Note that since we are mainly interested in corrections to scaling rather than in the leading scaling term, simulations for small system sizes play a significant role, and it is more important to achieve high-precision data for small and moderate values of *L* than going to even larger system sizes.

For systems with L≤256, more than $5\times 10^{7}$ statistically independent samples were generated, while for the system with L=1024, more than $5\times 10^{6}$ independent samples were generated.

## 5. Results

In this section, we discuss our findings. We have measured the quantities $C_{1}\left(L\right)$ and $S_{2}\left(L\right)$ on many finite lattices of linear size *L* and periodic boundary conditions. In order to study the thermodynamic limit (L→∞), we need to perform least-squares fits to the nonlinear FSS ansatz

(36) $$A\left(L\right)\;=\;L^{y_{A}}\;\left(a+b_{1}\;L^{-\omega }+b_{2}\;L^{-y_{2}}+b_{3}\;L^{-y_{3}}\right)$$

where $0<\omega <y_{2}<y_{3}$ are the correction-to-scaling exponents.

We have seen in Section 3, that for $A=C_{1}$, $y_{C_{1}}=d_{f}$, and for $A=S_{2}$, $y_{S_{2}}=2d_{f}-d=2d_{f}-2$. As the fractal dimension $d_{f}$ is known for the Potts model [cf. Equation (13)], we consider the reduced quantities

(37a) $$\tilde{C_{1}}=C_{1}L^{-d_{f}},$$

(37b) $$\tilde{S_{2}}=S_{2}L^{-(2d_{f}-2)}.$$

These quantities behave as follows:

(38) $$\tilde{A}\left(L\right)\;=\;a+b_{1}\;L^{-\omega }+b_{2}\;L^{-y_{2}}+b_{3}\;L^{-y_{3}}\;.$$

The parameter *a* gives the value of $\tilde{A}$ in the thermodynamic limit L→∞. The parameters $b_{i}$ represent the amplitudes of the corresponding FSS correction terms. The notation of this equation will be used when we discuss the numerical results of the MC simulations.

We have used Mathematica’s built-in function NonlinearModelFit to perform the weighted least-squares method for both linear and nonlinear fits to the ansatz (38). In order to detect corrections to scaling not taken into account in the ansatz (38), we have repeated each fit by only allowing data with $L\geq L_{\min}$. We then study the behavior of the estimated parameters as a function of $L_{\min}$. In general, our preferred fit will correspond to the smallest $L_{\min}$ for which the goodness of fit is reasonable, and for which subsequent increases in $L_{\min}$ do not cause the $\chi^{2}$ to drop *vastly* more than one unit per degree of freedom. For each fit, we report the observed value of the $\chi^{2}$, and the number of degrees of freedom (DF).

In the next sections, we will discuss the simulated models ordered in increasing value of the CG coupling *g*.

### 5.1. O(n) Loop Model on the Dense Branch $x_{-}$

In this subsection, we will discuss our results for the O(n) loop model on the dense branch $x_{-}$ with $n=1,\sqrt{2},\sqrt{3}$. These models belong to the same universality class as the critical Potts models with Q=1,2,3 states, respectively. The results are rather similar qualitatively, so we will discuss the first case in more detail and be brief in the other two cases.

#### 5.1.1. n=1

Let us start with the O(1) loop model on the dense branch, which reduces to the triangular-lattice site percolation and belongs to the same universality class as the critical one-state Potts model—i.e., the percolation universality. It is characterized by g=2/3, $d_{f}=91/48\approx 1.895833$, and $2d_{f}-2=43/24\approx 1.791667$.

In the least-squared fit, we include at most three correction terms as in Equation (38), and, furthermore, fix $y_{3}=2$ if not being explicitly specified. This is because, despite our extensive simulations, the precision of our data is not sufficient to simultaneously discern several correction terms. In addition, it seems unnecessary to include more rapidly decaying corrections.

We start with the reduced largest-cluster size ${\tilde{C}}_{1}$. The first step consists of fitting the data to a single power-law [i.e., we set $b_{2}=b_{3}=0$ in (38) so there are three free parameters $\left\{a,b_{1},\omega \right\}$]. We obtain a sensible fit for $L_{\min}=28$, giving ω=1.383(8) with $\chi^{2}/\mathrm{DF}=6.9/9$ (see first data row of Table 2). Even though the fit looks reasonable, the estimate for ω is significantly away from the expected result 1/g=3/2. We will show below that this is due to the effect of neglecting higher-order FSS corrections in the ansatz.

In a second step, we try to perform a fit to the ansatz (38) with $b_{3}=0$; i.e., with five free parameters $\left\{a,b_{1},b_{2},\omega ,y_{2}\right\}$. In this case, we do not obtain any good fit: either the program is unable to find a solution and/or the estimates for the parameters and their error bars take unreasonably large values. A similar behavior is found if we fix ω=1/g=3/2 and $b_{3}=0$ in the ansatz (38) so that there are four free parameters $\left\{a,b_{1},b_{2},y_{2}\right\}$. Again, the program is unable to converge or the estimates are unreasonably large. In other words, our data are not sufficiently accurate to simultaneously distinguish and determine two correction exponents whose values are rather close to each other. We refrain from including these failed fits in Table 2.

The third step involves performing a fit with two correction terms, where the subleading correction exponent is fixed. For $y_{2}=d_{f}=91/48$ and $b_{3}=0$, a good fit is achieved for $L_{\min}=12$ by using a four-parameter fit with $\left\{a,\omega ,b_{1},b_{2}\right\}$, yielding ω=1.50(1). Similarly, for $y_{3}=2$ and $b_{2}=0$, a good fit is obtained for $L_{\min}=16$ with a four-parameter fit involving $\left\{a,\omega ,b_{1},b_{3}\right\}$, resulting in ω=1.50(2) (see second and third data row on Table 2). Both fits show excellent agreement with the theoretical expectation 1/g=3/2. It is noted that the two amplitudes are similar in magnitude but have opposite signs in both fits.

**Figure 3:**
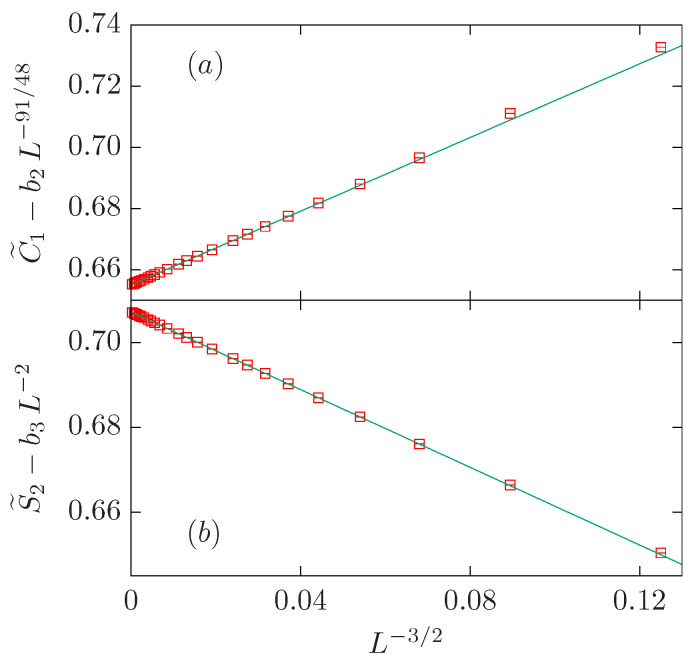
FSS corrections for the O(1) loop model on the dense branch, which belongs to the same universality class as the critical one-state Potts model. We show the quantities ${\tilde{C}}_{1}-b_{2}L^{-91/48}$ with $b_{2}=-0.582$ in panel (**a**), and ${\tilde{S}}_{2}-b_{3}L^{-2}$ with $b_{3}=0.679$ in panel (**b**). In both panels, data is plotted vs. $L^{-3/2}$. The smallest three system sizes are L=4,5,6, and the corresponding data points fall approximately on the straight line from the fits. This is a rather surprising fact.

To obtain further indication about which subleading correction term is somewhat more probable, we perform a fit with three correction terms for which all the correction exponents are fixed as ω=1/g=3/2, $y_{2}=91/48$ and $y_{3}=2$. Our preferred fit is obtained for $L_{\min}=12$. The amplitude $b_{3}$ is zero within error bars: i.e., $b_{3}=0.1\left(3\right)$. Therefore, we conclude that we need only two exponents ω=3/2 and $y_{2}=d_{f}=91/48$ to give account of the data with $L\geq L_{\min}=12$. The last row of the ${\tilde{C}}_{1}$ block in Table 2 displays the results of this final fit. These results are used in Figure 3a, which depicts the quantity ${\tilde{C}}_{1}-b_{2}L^{-91/48}$ vs. $L^{-3/2}$ where $b_{2}=-0.582$ is taken from the fit. According to our discussion, the data points form a straight line with a positive slope.

We now see why the first fits are not successful: there are two FSS corrections with exponents not too different (namely, 1/g=3/2=1.5 and $y_{2}=d_{f}=91/48\approx 1.895833$), and almost opposite amplitudes $b_{1}=0.602\left(2\right)$ vs. $b_{2}=-0.582\left(6\right)$. This is really a hard scenario for estimating these parameters, and some theoretical input is needed to disentangle these two contributions. Actually, they merge in such a way that they mimic a single power-law, like the one we obtained in the first step.

We will see that this subtle scenario persists for the second moment of the cluster sizes $S_{2}$ and for the other values of *n*.

In summary, the MC data for ${\tilde{C}}_{1}$ can be described with two FSS corrections: ω=1/g and $y_{2}=d_{f}$. The amplitude of the former (latter) correction term is positive (negative).

We can follow the same procedure for the ${\tilde{S}}_{2}$ data (see Table 2). If we set $b_{2}=b_{3}=0$, we obtain a good fit for $L_{\min}=24$ yielding ω=1.31(2). Again, this latter estimate is very far from the expected one 1/g=3/2.

If we fix $b_{2}$ or $b_{3}$ to 0 with $y_{2}=2d_{f}-2=43/24$ and $y_{3}=2$, we obtain sensible fits for $L_{\min}=7$. The corresponding estimates for ω are similar: 1.55(1) (1.50(1)) for $b_{3}=0$ ($b_{2}=0$). The corresponding amplitudes are positive, while $b_{1}$ is negative (see Table 2).

If ω=3/2, $y_{2}=2d_{f}-2$, and $y_{3}=2$ are fixed, with $b_{2}$ and $b_{3}$ treated as free parameters, we obtain a good fit for $L_{\min}=7$. It is worth noticing that the amplitude $b_{2}=0.00\left(8\right)$ is zero within errors, so we can safely assume $b_{2}=0$. Finally, if we fit the data to the ansatz (38) with ω=3/2, $y_{3}=2$, and $b_{2}=0$, we obtain a nice result for $L_{\min}=7$. In this case, the amplitudes $b_{1}$ and $b_{3}$ are again similar with opposite signs. Figure 3b shows the quantity ${\tilde{S}}_{2}-b_{3}L^{-2}$ vs. $L^{-3/2}$, and the data points form a straight line with a negative slope.

Therefore, the MC data can be described again with two FSS corrections: ω=1/g, and $y_{3}=2$. The amplitude of the former (latter) correction term is negative (positive).

#### 5.1.2. $n=\sqrt{2}$

The O($\sqrt{2})$ loop model on the dense branch belongs to the same universality class as the critical Ising model. They are characterized by g=3/4, $d_{f}=15/8=1.875$, and $2-2d_{f}=7/4=1.75$.

In this case, we have followed the procedure explained in Section 5.1.1; the results are displayed in Table 3.

The first step consists of a simple power-law fit to the ${\tilde{C}}_{1}$ data with $b_{2}=b_{3}=0$; the result for the exponent ω=1.269(8) is far away from the expected value 1/g=4/3 (see Table 3). Better results for ω are obtained if we fix $y_{2}=15/8$ or $y_{3}=2$. The fit with the three powers fixed to their expected values (ω=4/3, $y_{2}=15/8$, and $y_{3}=2$) is rather good already for $L_{\min}=9$. The value for the amplitude $b_{3}=0.27\left(8\right)$ is not far from zero (approximately three times the error bar), and it has the same sign as $b_{1}$ but the opposite sign as $b_{2}$. Since $b_{2}$ and $b_{3}$ can influence each other and potentially cancel out in the fit, we retain the larger correction term $b_{2}$ and set $b_{3}=0$ in the final fit. Notice that we obtain two similar amplitudes with opposite signs: $b_{1}=0.3724\left(7\right)$ and $b_{2}=-0.348\left(3\right)$.

The analysis of the ${\tilde{S}}_{2}$ data follows the same procedure. In this case, the expected value for ω is very well reproduced if we fix $b_{2}=0$ and $y_{3}=2$; namely, ω=1.332(4). In the three-parameter fit with all powers fixed to their expected values, it is clear that $b_{2}=0$ within error. Therefore, our preferred fit corresponds to the last row of the second block in Table 3. Again, we find two similar amplitudes with opposite signs: $b_{1}=-0.3098\left(3\right)$ and $b_{3}=0.496\left(1\right)$.

**Figure 4:**
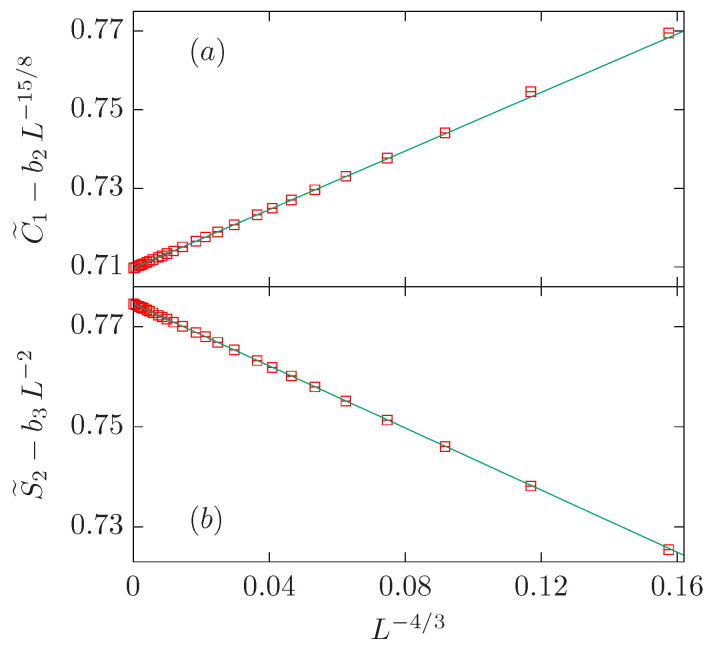
FSS corrections for the O$\left(\sqrt{2}\right)$ loop model on the dense branch, which has the same universality class as the critical Ising model. We show the quantities ${\tilde{C}}_{1}-b_{2}L^{-15/8}$ with $b_{2}=-0.348$ in panel (**a**), and ${\tilde{S}}_{2}-b_{3}L^{-2}$ with $b_{3}=0.496$ in panel (**b**). In both panels, data is represented vs. $L^{-4/3}$. It can be clearly seen that the coefficient of the leading correction, with exponent −1/g, has opposite signs for $C_{1}$ and $S_{2}$, suggesting that the small clusters contribute more than the largest one. This scenario holds true for the whole dense branch till the O(2) case, where both contributions cancel each other.

In summary, we have found that both data sets can be well described with two powers with exponents 1/g=4/3 and $y_{2}=d_{f}$ ($y_{3}=2$) for the ${\tilde{C}}_{1}$ (${\tilde{S}}_{2}$) data set. In both cases, we find two similar amplitudes with opposite signs (see Figure 4).

Finally, it is worth noting that the correction exponent 1/g=4/3 appears only when considering geometric objects in the Ising model. In fact, for the Ising model in the spin representation [8], one only finds integer exponents: i.e., ω=2. This is exactly the expected behavior [19,20,61,62].

#### 5.1.3. $n=\sqrt{3}$

The O$\left(\sqrt{3}\right)$ loop model on the dense branch belongs to the same universality class as the critical three-state Potts model. Both models are characterized by g=5/6, $d_{f}=28/15\approx 1.866667$, and $2d_{f}-2=26/15\approx 1.733333$.

We have followed the procedure outlined in Section 5.1.1; the results are displayed on the last two blocks of Table 3.

Again, if we fit the ${\tilde{C}}_{1}$ data to a simple power-law with $b_{2}=b_{3}=0$, we obtain a result for the exponent ω=1.141(6) which is far away from the expected value 1/g=6/5 (see Table 3). Better results for ω are obtained if we fix $y_{2}=28/15$ or $y_{3}=2$. The fit with the three powers fixed to their expected values (ω=6/5, $y_{2}=28/15$, and $y_{3}=2$) is rather good already for $L_{\min}=10$. The value for the amplitude $b_{3}=0.0\left(1\right)$ agrees with zero within error, so we set it to $b_{3}=0$ in the last fit. We obtain two similar amplitudes with opposite signs: $b_{1}=0.2569\left(7\right)$ and $b_{2}=-0.226\left(3\right)$.

The analysis of the ${\tilde{S}}_{2}$ data follows the same procedure. In this case, the expected value for ω is very well reproduced if we fix $b_{2}=0$ and $y_{3}=2$. In the three-parameter fit with all powers fixed to their expected values, it is clear that $b_{2}=0$ within error bars. Therefore, our preferred fit corresponds to $b_{1}=-0.1831\left(5\right)$ and $b_{3}=0.338\left(3\right)$ (see Table 3). They are quite similar with opposite signs.

**Figure 5:**
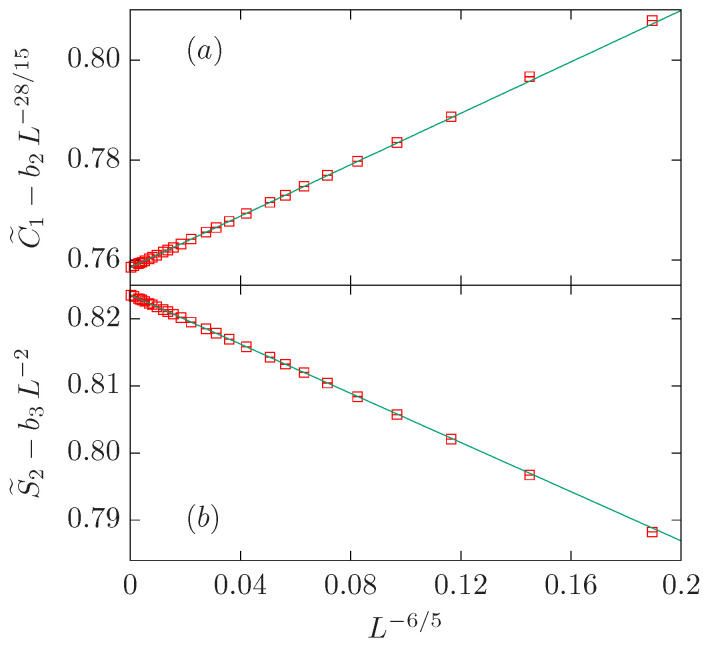
FSS corrections for the O$\left(\sqrt{3}\right)$ loop model on $x_{-}$, which belongs to the same universality class as the critical three-state Potts model. We show the quantities ${\tilde{C}}_{1}-b_{2}L^{-28/15}$ with $b_{2}=-0.226$ in panel (**a**) and ${\tilde{S}}_{2}-b_{3}L^{-2}$ with $b_{3}=0.338$ in panel (**b**). In both panels, data is depicted vs. $L^{-6/5}$.

In summary, we have found that both data sets can be well described with two powers with exponents 1/g=6/5 and $y_{2}=d_{f}$ ($y_{3}=2$) for the ${\tilde{C}}_{1}$ (${\tilde{S}}_{2}$) data set. In both cases, we find two similar amplitudes with opposite signs (see Figure 5).

### 5.2. O(2) Loop Model

The O(2) loop model belongs to the same universality class as the critical four-state Potts model. Both models are characterized by g=1, $d_{f}=15/8=1.875$, and $2d_{f}-2=7/4=1.75$. This model corresponds to the point where the dense and dilute branches meet $x_{-}=x_{+}$.

We have followed the procedure outlined in Section 5.1.1; the results are displayed in Table 4.

In this case, one might expect logarithmic correction due to the existence of the marginally irrelevant dilution field (i.e., $y_{t2}=0$) [13,14,15]. As observed in Ref. [34], simulating the equivalent O(2) loop model makes these logarithmic corrections to disappear.

**Figure 6:**
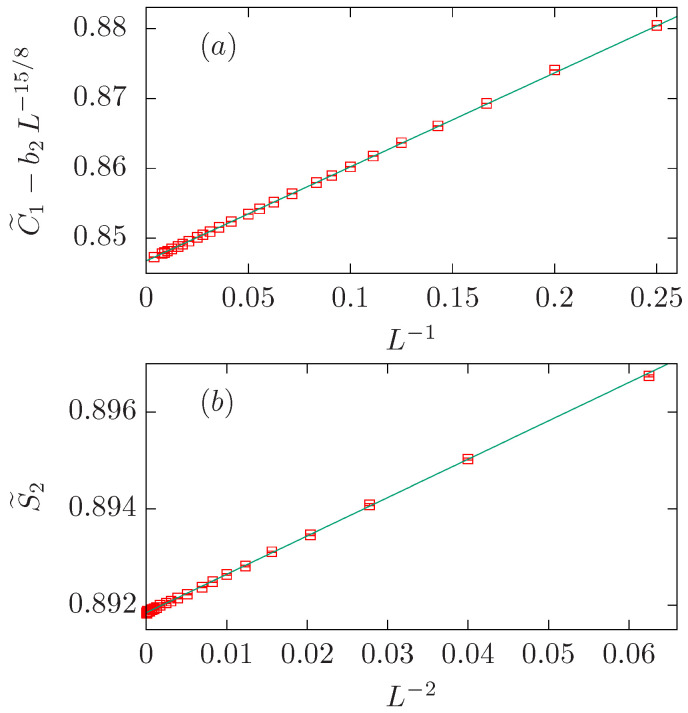
FSS corrections for the O(2) loop model, which belongs to the same universality class as the critical four-state Potts model. We show the quantities ${\tilde{C}}_{1}-b_{2}L^{-15/8}$ vs. $L^{-1}$ with $b_{2}=-0.082$ in panel (**a**), and ${\tilde{S}}_{2}$ vs. $L^{-2}$ in panel (**b**). It is clearly seen that the theoretically predicted correction term, with exponent −1, is absent in $S_{2}$. This effect can be attributed to the exact cancellation of the contributions from large clusters and from the smaller ones.

The fit to the data ${\tilde{C}}_{1}$ is straightforward. We find that the dominant exponent is 1/g=1, while the subdominant exponent is $d_{f}=15/8$ [see Table 4 and Figure 6a].

On the other hand, the fit to the data ${\tilde{S}}_{2}$ presents some surprises. We first observe that the fit to a single power-law (i.e., setting $b_{2}=b_{3}=0$) gives a power ω=2.01(5) with a small amplitude $b_{1}=0.079\left(9\right)$. Moreover, the fits with $y_{2}=d_{f}$, $b_{3}=0$ or with $b_{2}=0$, $y_{3}=2$ give useless results: either the procedure does not converge, or the error bars are extremely large. If we set ω=1/g=1, $y_{2}=d_{f}$, and $y_{3}=2$, we find an extremely small value for the amplitude $b_{1}$. Furthermore, if we set $b_{1}=0$, $y_{2}=d_{f}$, and $y_{3}=2$, the subsequent fit shows that $b_{2}=0$ within statistical errors. Therefore, for this case, we find that the dominant FSS term corresponds to $y_{3}=2$ with a small amplitude $b_{3}$ [see Table 4 and Figure 6b].

### 5.3. O(n) Loop Model on the Dilute Branch $x_{+}$

In this section, we will discuss our results for the O(n) loop model on the dilute branch $x_{+}$ with $n=\sqrt{3},\sqrt{2},1$, which belong to the universality classes of the tricritical Potts model with Q=3,2,1 states, respectively. The results are rather similar qualitatively, so we will discuss the first case in more detail and be brief in the other two cases.

#### 5.3.1. $n=\sqrt{3}$

The O$\left(\sqrt{3}\right)$ loop model on the dilute branch belongs to the same universality class as the tricritical three-state Potts model. Both models are characterized by g=7/6, $d_{f}=40/21\approx 1.904762$, $2d_{f}-2=38/21\approx 1.809524$, and 4/g−2=10/7≈1.428571.

Let us start with the fit of the ${\tilde{C}}_{1}$ data. If we fit the data to a single power-law ansatz [e.g., (38) with $b_{2}=b_{3}=0$], we obtain an estimate ω=0.838(5) which is far (i.e., four standard deviations) from the expected result 1/g=6/7≈0.857143. Better estimates are obtained if we set $y_{2}=d_{f}=40/21$ and $b_{3}=0$, or $b_{2}=0$ and $y_{3}=2$. If we set ω=1/g=6/7, $y_{2}=d_{f}$, and $y_{3}=2$, we obtain a good estimate for $L_{\min}=6$. It is worth noting that the estimates for both $b_{2}$ and $b_{3}$ are compatible with zero within two standard deviations. Finally, we set ω=1/g, and $b_{2}=b_{3}=0$. The fit is reasonable for $L_{\min}=20$ with a small (but nonzero) amplitude $b_{1}$.

Dealing with the ${\tilde{S}}_{2}$ is now a bit more complicated than for its dense-branch counterpart, as there is one extra candidate that may play a role; namely, the exponent 4/g−2=10/7. As before, we start with a single power-law fit [$b_{2}=b_{3}=0$ in (38)]. The result is ω=0.827(7), which is again many standard deviations from the expected result. Better results on average can be obtained by fixing one correction-to-scaling exponent: either $y_{2}=2d_{f}-2$, $y_{2}=4/g-2$, or $y_{3}=2$. It is striking that these fits are already excellent for $L_{\min}=4$. If we now fix ω=1/g=6/7, $y_{3}=2$, and either $y_{2}=2d_{f}-2$ or $y_{2}=4/g-2$, we obtain good fits with the amplitude $b_{2}=0$ within errors. The amplitude $b_{3}$ seems to be small but nonzero. We can check this observation by fixing ω=1/g, $b_{2}=0$, and $y_{3}=2$. The result of such a fit confirms the previous observation. It is approximately half of $b_{1}$ with the opposite sign.

**Figure 7:**
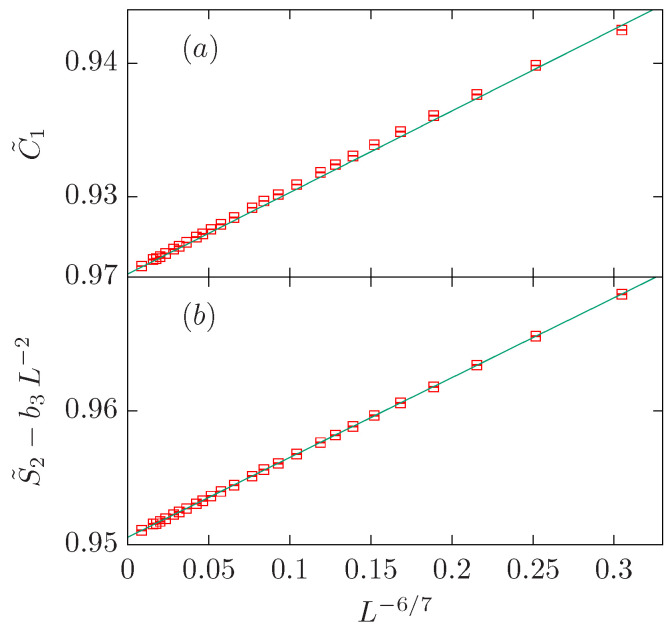
FSS corrections for the O$\left(\sqrt{3}\right)$ loop model on the dilute branch $x_{+}$, which belongs to the same universality class as the tricritical three-state Potts model. We show the quantities ${\tilde{C}}_{1}$ in panel (**a**) and ${\tilde{S}}_{2}-b_{3}L^{-2}$ with $b_{3}=-0.028$ in panel (**b**). In both panels, data points are depicted vs. $L^{-6/7}$.

In summary, we have found that the ${\tilde{C}}_{1}$ data set can be well described with a single power with exponent 1/g=6/7. The amplitude $b_{1}$ is rather small compared to *a*. On the other hand, the ${\tilde{S}}_{2}$ data set can be described with two exponents 1/g=6/7 and $y_{3}=2$. The amplitudes $b_{1}$ and $b_{3}$ are rather similar in absolute value but have opposite signs. We have already found this situation in the study of the dense branch. These two scenarios are depicted in Figure 7.

#### 5.3.2. $n=\sqrt{2}$

The O$\left(\sqrt{2}\right)$ loop model on the dilute branch belongs to the same universality class as the tricritical Ising model (Q=2). Both models are characterized by g=5/4, $d_{f}=77/40=1.925$, $2d_{f}-2=37/20=1.85$, and 4/g−2=6/5=1.2.

**Figure 8:**
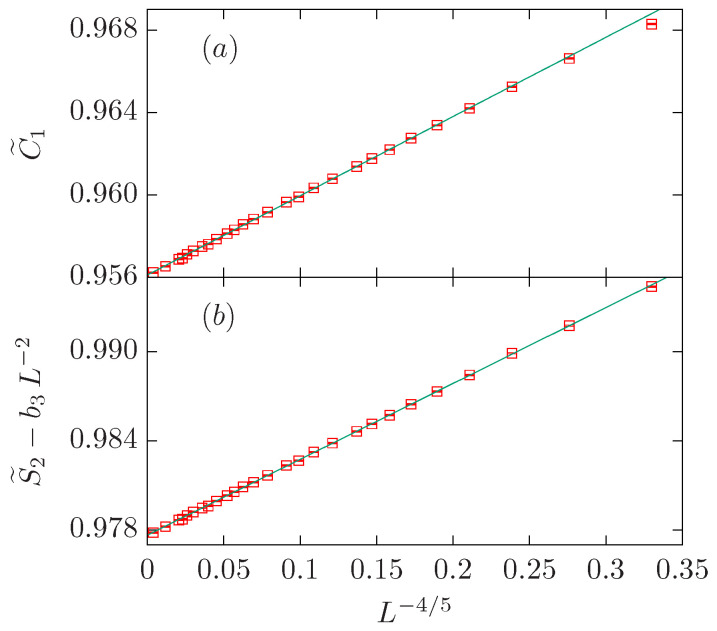
FSS corrections for the O$\left(\sqrt{2}\right)$ loop model on the dilute branch, which belongs to the same universality class as the tricritical Ising model. We show the quantities ${\tilde{C}}_{1}$ in panel (**a**) and ${\tilde{S}}_{2}-b_{3}L^{-2}$ with $b_{3}=-0.039$ in panel (**b**). In both panels, we depict the data points vs. $L^{-4/5}$.

Let us start with the fit of the ${\tilde{C}}_{1}$ data. If we fit the data to a single power-law ansatz [i.e., $b_{2}=b_{3}=0$ in the ansatz (38)], we obtain an estimate ω=0.779(4) which is five standard deviations away from the expected result 1/g=4/5=0.8 (see Table 5). If we set $y_{2}=d_{f},b_{3}=0$ or $y_{3}=2,b_{2}=0$, we obtain a similar result for ω, as the estimates for $b_{2}$ or $b_{3}$ are compatible with zero within error bars. Therefore, we refit the data assuming that $b_{2}=b_{3}=0$, and obtain a good fit for a larger value of $L_{\min}=20$. The plot of ${\tilde{C}}_{1}$ vs. $L^{-4/5}$ is depicted in Figure 8a.

The fit to the ${\tilde{S}}_{2}$ data is similar. The initial fit to a simple power law (see Table 5) shows an stable fit for $L_{\min}=20$ which yields an estimate for ω=0.79(2) that is compatible within error bars with the expected result 1/g=4/5=0.8. The fits with either $b_{3}=0,y_{2}=2d_{f}-2,4/g-2$ or $b_{2}=0,y_{3}=2$ fixed give estimates for ω that are compatible within error bars with the expected result. It is worth noticing that the fit with $y_{2}=4/g-2=6/5$ is rather unstable numerically. The amplitudes $b_{2}$ and $b_{3}$ are rather small. We chose $b_{2}=0$ in our final estimate as in previous cases. The data is displayed in Figure 8b.

#### 5.3.3. n=1

The O(1) loop model on the dilute branch $x_{+}$ is just the triangular-lattice Ising model, and the clusters studied hereby are the Ising domains. The critical behavior of these Ising domains is expected to belong to the same universality class as the tricritical one-state Potts model. Both models are characterized by g=4/3, $d_{f}=187/96\approx 1.9479167$, $2d_{f}-2=91/48\approx 1.895833$, and 4/g−2=1.

**Figure 9:**
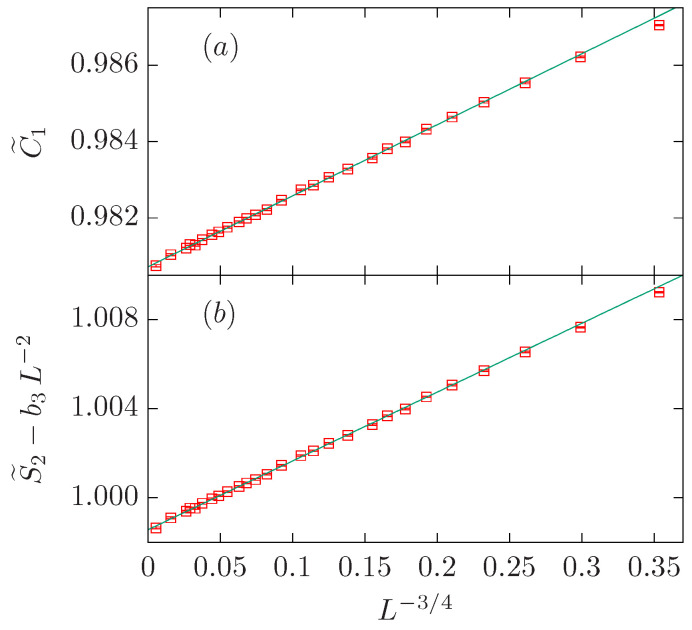
FSS corrections for the O(1) loop model on the dilute branch, which belongs to the same universality class as the tricritical one-state Potts model. We show the quantities ${\tilde{C}}_{1}$ in panel (**a**) and ${\tilde{S}}_{2}-b_{3}L^{-2}$ with $b_{3}=-0.032$ in panel (**b**). In both panels, data points are depicted vs. $L^{-3/4}$.

Let us start with the fit of the ${\tilde{C}}_{1}$ data. If we fit the data to a single power-law ansatz [i.e., $b_{2}=b_{3}=0$ in the ansatz (38)], we obtain an estimate ω=0.72(2) which is 1.5 standard deviations away from the expected result 1/g=3/4=0.75 (see Table 5). If we set $y_{2}=d_{f},b_{3}=0$ or $y_{3}=2,b_{2}=0$, we obtain a similar result for ω, as the estimates for $b_{2}$ or $b_{3}$ are compatible with zero within error bars. Therefore, we refit the data assuming that $b_{2}=b_{3}=0$, and obtain a good fit for a larger value of $L_{\min}=14$. The plot of ${\tilde{C}}_{1}$ vs. $L^{-3/4}$ is depicted in Figure 9a.

The fit to the ${\tilde{S}}_{2}$ data is similar. The initial fit to a simple power law (see Table 5) shows an stable fit for $L_{\min}=14$, which yields an estimate for ω=0.70(2) that is 2.5 standard deviations from the expected result 1/g=3/4=0.75. The fits with either $b_{3}=0,y_{2}=2d_{f}-2$ or $b_{2}=0,y_{3}=2$ give estimates for ω that are compatible within error bars with the expected result. It is worth noticing that the fit with $y_{2}=4/g-2=1$ is very unstable numerically and has not been included in Table 5. The amplitudes $b_{2}$ and $b_{3}$ are rather small. We chose $b_{2}=0$ in our final estimate as in previous cases. The data is displayed in Figure 9b.

Notice that the O(1) loop model on the dilute branch has the same conformal charge as the critical Ising model (c=1/2). However, since we study the critical behavior of the geometric Ising domains instead of the conventional thermodynamic quantities, the FSS corrections are very different, as we have seen in this section.

## 6. Discussion

We have studied the FSS corrections for geometric observables in the O(n) loop model on both the dense and dilute branches. These models belong to the same universality classes as the critical and tricritical Potts models with $Q=n^{2}$. We have found that the leading correction term has an exponent given by 1/g, in agreement with previous studies [43,44]. We have also found that for the quantity $C_{1}$, the second correction term has an exponent $d_{f}$. For the quantity $S_{2}$, the subleading correction term has an exponent 2. No numerical evidence is obtained for the predicted subleading correction exponent 4/g−2 for the dilute branch $x_{+}$ of the O(n) loop model (i.e., the tricritical $Q=n^{2}$ Potts model), which might be due to some symmetries in the O(n) loop model.

**Figure 10:**
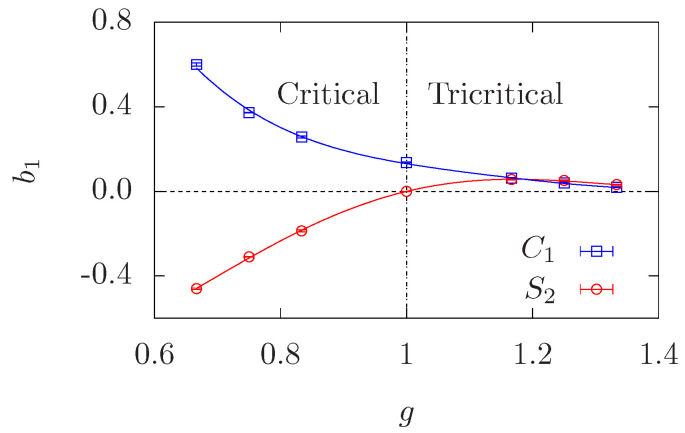
Coefficient $b_{1}$ associated to the term $L^{-\omega }$ with ω=1/g in the ansatz (38). We display the numerical results for $b_{1}$ obtained from the fits to $C_{1}$ (blue curve) and $S_{2}$ (red curve). Curves are only to guide the eye.

We have also studied the amplitudes of the corresponding terms. In general, we have found that the amplitudes of the leading and subleading terms are similar and with opposite signs. This is the reason why extracting these exponents has been very elusive in previous studies. The amplitude of the leading term $b_{1}$ is the one with the smallest error bars, so we can draw some firmer conclusions. In Figure 10, we have plotted the amplitude $b_{1}$ as a function of the CG coupling *g* for all the models we have considered in this paper. For $C_{1}$ (blue curve in Figure 10), $b_{1}$ is positive and seems to be a decreasing function of *g* in the interval g∈[2/3,4/3], ranging from $b_{1}=0.602\left(2\right)$ for g=2/3 to $b_{1}=0.0187\left(2\right)$ for g=4/3.

On the other hand, the amplitude $b_{1}$ for $S_{2}$ (red curve in Figure 10) is negative for g∈[2/3,1), vanishes at g=1, and becomes positive and small for g∈(1,4/3]. In this latter regime, it is quite similar to the amplitude $b_{1}$ for $C_{1}$. We recall here the fact (explained in Section 3) that the amplitude $b_{1}$ for $S_{2}$ has contributions from the amplitude $b_{1}$ for $C_{1}$ (large clusters) and from the size distribution $n(s,p_{c})$ (small clusters). This picture explains the behavior shown in Figure 10: the red curve contains a (negative) contribution from small clusters and a (positive) contribution from large clusters (which is given by the blue curve). As *g* increases, these two contributions become to approximate in absolute value, and exactly at Q=4, they compensate so $b_{1}=0$ for $S_{2}$. The amplitude $b_{2}$ of the subleading term is similar to and with opposite sign than $b_{1}$. Moreover, $b_{2}$ for $C_{1}$ is zero for the tricritical Potts models.

Our study demonstrates the existence of the subleading magnetic scaling field in both the finite-cluster-size and finite-system-size corrections. It delivers a warning message that, in FSS analysis of the Potts model, the frequently ignored contributions from the subleading magnetic field might play a non-negligible contributions. It would be interesting to extend our studies for the percolation and the Ising model in three and higher dimensions, where little knowledge has been known so far.

## Figures and Tables

**Table 1:** Critical exponents for the critical and tricritical *Q*-state Potts models. For each model and value of *Q*, we show the CG coupling *g*, the dominant thermal exponents $y_{t1}$ (22a), the subdominant thermal exponent $y_{t2}$ (22b), the dominant magnetic exponent $y_{h1}$ (23) [which is equal to the fractal dimension $d_{f}$], the subdominant magnetic exponent $y_{h2}$ (23), and their difference $y_{h1}-y_{h2}=1/g$.

		Critical				Tricritical		
		Q=1	Q=2	Q=3	Q=4	Q=3	Q=2	Q=1
	*g*	2/3	3/4	5/6	1	7/6	5/4	4/3
$y_{t1}$	3−3/2g	3/4	1	6/5	3/2	12/7	9/5	15/8
$y_{t2}$	4−4/g	−2	−4/3−	−4/5−	0	4/7	4/5	1
$y_{h1}=d_{f}$	(2g+1)(2g+3)/8g	91/48	15/8	28/15	15/8	40/21	77/40	187/96
$y_{h2}$	(2g−1)(2g+5)/8g	19/48	13/24	2/3	7/8	22/21	9/8	115/96
$y_{h1}-y_{h2}$	1/g	3/2	4/3	6/5	1	6/7	4/5	3/4

**Table 2:** FSS corrections for the O(1) loop model on the dense branch $x_{-}$, which belongs to the same universality class as the critical one-state Potts model. Both models are characterized by g=2/3, $d_{f}=91/48$, and $2d_{f}-2=43/24$. We show the fits of the quantities ${\tilde{C}}_{1}$ and ${\tilde{S}}_{2}$ to the full ansatz (38).

	Fits for n=1 on the Dense Branch $x_{-}$								
$\tilde{O}$	$L_{\min}$	a	$b_{1}$	ω	$b_{2}$	$y_{2}$	$b_{3}$	$y_{3}$	$\chi^{2}/\mathrm{DF}$
${\tilde{C}}_{1}$	28	0.655033(7)	0.304(9)	1.383(9)	0		0		6.9/9
	12	0.655045(6)	0.60(4)	1.50(1)	−0.57(6)	91/48	0		10.0/14
	16	0.655052(8)	0.58(7)	1.50(2)	0		−0.7(1)	2	7.1/12
	12	0.655045(6)	0.61(2)	3/2	−0.6(3)	91/48	0.1(3)	2	10.0/14
	12	0.655046(3)	0.602(2)	3/2	−0.582(6)	91/48	0		10.0/15
${\tilde{S}}_{2}$	24	0.70729(1)	−0.175(10)	1.31(2)	0		0		7.5/10
	7	0.707245(9)	−0.67(4)	1.55(1)	0.87(5)	43/24	0		12.0/19
	7	0.707251(9)	−0.46(2)	1.50(1)	0		0.68(3)	2	11.4/19
	7	0.707250(8)	−0.46(2)	3/2	0.00(8)	43/24	0.68(7)	2	11.4/19
	7	0.707250(5)	−0.458(1)	3/2	0		0.679(3)	2	11.4/20

**Table 3:** FSS corrections for the O($\sqrt{2})$ and O($\sqrt{3})$ loop models on the dense branch, which belong to the same universality classes as the critical Ising model (Q=2) and the three-state Potts models, respectively. The former is characterized by g=3/4, $d_{f}=15/8$, and $2d_{f}-2=7/4$, and the latter is given by g=5/6, $d_{f}=28/15$, and $2d_{f}-2=26/15$. We show the fits of the quantities ${\tilde{C}}_{1}$ and ${\tilde{S}}_{2}$ to the full ansatz (38).

Fits for the Dense Branch $x_{-}$										
n	$\tilde{O}$	$L_{\min}$	a	$b_{1}$	ω	$b_{2}$	$y_{2}$	$b_{3}$	$y_{3}$	$\chi^{2}/\mathrm{DF}$
$\sqrt{2}$	${\tilde{C}}_{1}$	40	0.709702(6)	0.258(8)	1.269(8)	0		0		0.56/7
		9	0.709702(5)	0.346(8)	1.317(5)	−0.30(1)	15/8	0		10.3/18
		10	0.709701(5)	0.316(7)	1.304(5)	0		−0.31(2)	2	6.9/17
		9	0.709703(4)	0.383(4)	4/3	−0.59(7)	15/8	0.27(8)	2	8.3/18
		10	0.709711(3)	0.3724(7)	4/3	−0.348(3)	15/8	0		9.5/18
	${\tilde{S}}_{2}$	28	0.774568(9)	−0.179(6)	1.22(1)	0		0		6.3/10
		12	0.774550(9)	−0.47(5)	1.39(2)	0.55(6)	7/4	0		7.0/15
		6	0.774550(6)	−0.309(4)	1.332(4)	0		0.494(7)	2	15.2/21
		5	0.774552(5)	−0.313(3)	4/3	0.02(1)	7/4	0.47(1)	2	16.7/22
		6	0.774548(3)	−0.3098(3)	4/3	0		0.496(1)	2	15.3/22
$\sqrt{3}$	${\tilde{C}}_{1}$	24	0.758498(9)	0.192(3)	1.141(6)	0		0		12.6/11
		10	0.758516(9)	0.258(9)	1.201(9)	−0.23(3)	28/15	0		14.8/17
		10	0.758512(9)	0.237(7)	1.186(8)	0		−0.23(2)	2	15.4/17
		10	0.758515(8)	0.257(4)	6/5	−0.2(1)	28/15	0.0(1)	2	14.9/17
		10	0.758515(5)	0.2569(7)	6/5	−0.226(3)	28/15	0		14.9/18
	${\tilde{S}}_{2}$	24	0.82358(1)	−0.110(3)	1.084(9)	0		0		12.9/11
		7	0.823531(9)	−0.265(9)	1.257(8)	0.35(1)	26/15	0		16.5/20
		9	0.82354(1)	−0.177(6)	1.191(9)	0		0.32(2)	2	12.9/18
		9	0.82354(8)	−0.187(4)	6/5	0.04(4)	26/15	0.29(5)	2	13.1/18
		9	0.823532(5)	−0.1831(5)	6/5	0		0.338(3)	2	13.9/19

**Table 4:** FSS corrections for the O(2) loop model and the O$\left(\sqrt{3}\right)$ loop model on the dilute branch $x_{+}$, which belong to the same universality classes as the critical four-state Potts and the tricritical three-state Potts models, respectively. The former model is characterized by g=1, $d_{f}=15/8$, and $2d_{f}-2=7/4$, and the latter one, by g=7/6, $d_{f}=40/21$, $2d_{f}-2=38/21$, and 4/g−2=10/7. We show the fits of the quantities ${\tilde{C}}_{1}$ and ${\tilde{S}}_{2}$ to the full ansatz (38).

$\tilde{O}$	$L_{\min}$	*a*	$b_{1}$	ω	$b_{2}$	$y_{2}$	$b_{3}$	$y_{3}$	$\chi^{2}/\mathrm{DF}$
Fits for the O(2) loop model									
${\tilde{C}}_{1}$	24	0.84673(3)	0.120(4)	0.97(1)	0		0		10.4/10
	7	0.84673(2)	0.127(4)	0.98(1)	−0.06(1)	15/8	0		18.4/19
	7	0.84673(2)	0.124(3)	0.979(9)	0		−0.06(1)	2	18.5/19
	8	0.84674(2)	0.137(2)	1	−0.3(1)	15/8	0.3(1)	2	16.2/18
	10	0.84676(1)	0.1345(6)	1	−0.082(5)	15/8	0		15.5/17
${\tilde{S}}_{2}$	9	0.891851(4)	0.079(9)	2.01(5)	0		0		16.1/18
	8	0.89183(1)	0.003(1)	1	−0.09(3)	7/4	0.20(5)	2	13.5/18
	9	0.891850(4)	0		0.000(8)	7/4	0.08(1)	2	16.1/18
	9	0.891850(3)	0		0		0.0775(9)	2	16.1/19
Fits for the O$\left(\sqrt{3}\right)$ loop model on the dilute branch $x_{+}$									
${\tilde{C}}_{1}$	8	0.92423(3)	0.0610(6)	0.838(5)	0		0		14.6/19
	6	0.92431(4)	0.069(3)	0.88(1)	−0.029(6)	40/21	0		11.6/20
	6	0.92430(4)	0.068(2)	0.87(1)	0		−0.031(8)	2	11.6/20
	6	0.92428(2)	0.064(1)	6/7	0.1(1)	40/21	−0.2(1)	2	14.7/20
	20	0.92428(1)	0.0639(4)	6/7	0		0		5.52/12
${\tilde{S}}_{2}$	8	0.95050(3)	0.0542(7)	0.827(7)	0		0		13.2/19
	4	0.95059(3)	0.064(2)	0.88(1)	−0.031(4)	38/21	0		10.6/22
	4	0.95063(4)	0.080(5)	0.93(2)	−0.043(7)	10/7	0		11.1/22
	4	0.95058(3)	0.061(2)	0.87(1)	0		−0.031(4)	2	10.6/22
	4	0.95056(2)	0.0590(8)	6/7	0.02(2)	38/21	−0.05(3)	2	10.7/22
	4	0.95057(2)	0.059(1)	6/7	0.006(7)	10/7	−0.036(9)	2	10.6/22
	4	0.95055(1)	0.0597(2)	6/7	0		−0.028(1)	2	11.4/23

**Table 5:** FSS corrections for the O$\left(\sqrt{2}\right)$ and O(1) loop models on the dilute branch, which belong to the same universality class as the tricritical Ising model (Q=2) and the one-state tricritical Potts model, respectively. The former is characterized by g=5/4, $d_{f}=77/40$, $2d_{f}-2=37/20$, and 4/g−2=6/5. The latter is given by g=4/3, $d_{f}=187/96$, $2d_{f}-2=91/48$, and 4/g−2=1. We show the fits of the quantities ${\tilde{C}}_{1}$ and ${\tilde{S}}_{2}$ to the full ansatz (38).

Fits for the Dilute Branch $x_{+}$										
n	$\tilde{O}$	$L_{\min}$	a	$b_{1}$	ω	$b_{2}$	$y_{2}$	$b_{3}$	$y_{3}$	$\chi^{2}/\mathrm{DF}$
$\sqrt{2}$	${\tilde{C}}_{1}$	5	0.95603(2)	0.0372(3)	0.779(4)	0		0		15.4/23
		5	0.95604(3)	0.037(1)	0.78(1)	0.00(4)	77/40	0		15.4/22
		5	0.95604(3)	0.037(1)	0.78(1)	0		0.00(4)	2	15.4/22
		5	0.95606(2)	0.0401(6)	4/5	−0.13(8)	77/40	0.13(9)	2	15.4/22
		20	0.95606(2)	0.0396(4)	4/5	0		0		9.60/13
	${\tilde{S}}_{2}$	20	0.97761(5)	0.050(3)	0.79(2)	0		0		10.4/12
		5	0.97760(4)	0.051(2)	0.79(1)	−0.027(5)	37/20	0		16.8/22
		8	0.97763(6)	0.08(2)	0.86(5)	−0.04(2)	6/5	0		13.7/19
		5	0.97759(4)	0.049(2)	0.79(1)	0		−0.029(6)	2	16.9/22
		6	0.97759(3)	0.053(1)	4/5	−0.14(7)	37/20	0.14(9)	2	14.3/21
		6	0.97758(3)	0.055(2)	4/5	−0.013(8)	6/5	−0.01(1)	2	15.0/21
		7	0.97762(2)	0.0516(4)	4/5	0		−0.039(4)	2	15.6/21
1	${\tilde{C}}_{1}$	10	0.98067(3)	0.0174(6)	0.72(2)	0		0		16.4/18
		5	0.98069(4)	0.018(1)	0.73(2)	0.000(4)	187/96	0		21.2/22
		5	0.98069(3)	0.018(1)	0.73(2)	0		0.000(4)	2	21.2/22
		8	0.98068(3)	0.0200(9)	3/4	−0.5(3)	187/96	0.5(3)	2	18.8/19
		14	0.98071(1)	0.0187(2)	3/4	0		0		15.7/16
	${\tilde{C}}_{1}$	14	0.99850(6)	0.027(1)	0.70(2)	0		0		13.6/15
		6	0.99854(6)	0.030(2)	0.73(3)	−0.021(7)	91/48	0		21.1/21
		4	0.99857(5)	0.032(1)	0.75(2)	0		−0.033(4)	2	22.8/22
		4	0.99858(3)	0.0307(6)	3/4	0.04(5)	91/48	−0.07(6)	2	22.3/23
		4	0.99858(4)	0.030(2)	3/4	0.001(4)	1	−0.034(5)	2	22.7/23
		4	0.99856(2)	0.0312(2)	3/4	0		−0.032(1)	2	22.8/24

## Data Availability

The raw data supporting the conclusions of this article will be made available by the authors on request.

## Abbreviations

The following abbreviations are used in this manuscript:

MC	Monte Carlo
FSS	Finite size scaling
RG	Renormalization group
2D	Two dimensional
CFT	Conformal field theory
FK	Fortuin Kasteleyn
CG	Coulomb gas
CSD	Critical slowing down
DF	Degrees of freedom
